# Learning in a Pandemic: Primary School children’s Emotional Engagement with Remote Schooling during the spring 2020 Covid-19 Lockdown in Ireland

**DOI:** 10.1007/s12187-022-09922-8

**Published:** 2022-02-12

**Authors:** Yekaterina Chzhen, Jennifer Symonds, Dympna Devine, Júlia Mikolai, Susan Harkness, Seaneen Sloan, Gabriela Martinez Sainz

**Affiliations:** 1 Trinity College Dublin, Department of Sociology, Dublin, Ireland; 2 University College Dublin, School of Education, Dublin, Ireland; 3 University of St Andrews, School of Geography and Sustainable Development, St Andrews, UK; 4 University of Bristol, School of Policy Studies, Bristol, UK

**Keywords:** COVID-19, School closures, Remote schooling, Engagement with schooling, Ireland

## Abstract

The COVID-19 pandemic resulted in the greatest disruption to children’s schooling in generations. This study analyses primary school children’s emotional engagement with remote schooling during the Spring 2020 lockdown in the Republic of Ireland, which involved one of the longest school closures among rich countries at the time. It investigates whether children’s engagement with their remote schooling varied by personal and family characteristics, using data from the Children’s School Lives (CSL) surveys. CSL is a nationally representative study of primary schools in Ireland, which collected information from children aged 8–9 years in May – August 2019 and in May – July 2020. Linear regression estimates with school fixed effects are based on the analytic sample of nearly 400 children (from across 71 schools) who took part in both waves and have complete data on all the key variables. Emotional engagement with schooling is measured using child-reported items on satisfaction with schooling. Everything else being equal, children who reported higher engagement with schooling before the pandemic were more engaged with remote schooling during the lockdown. Although there were no significant differences by family affluence, children with greater resources for home schooling reported higher levels of engagement. This includes having a computer or a laptop for schoolwork, having someone to help with schoolwork if the child is worried about falling behind, and having schoolwork checked by a teacher. This points to the paramount importance of adequate digital technologies in the home as well as the availability of help during periods of remote schooling.

## Introduction

The COVID-19 pandemic is perhaps the biggest global crisis of this century. In addition to the direct health effects of the outbreak, measures to halt its spread have put economies on hold and disrupted every sphere of life. School closures have been a key component of virus containment measures in many countries at the start of the pandemic. In 2020, schools were closed for an average of four months worldwide and an estimated 24 million children and young people globally were at risk of not returning to learning due to the economic impacts of the COVID-19 crisis (UNESCO, 2021). In February 2021, one year into the pandemic, primary schools were open for in-school (rather than remote or ‘hybrid’) learning for the majority of their students in only 30% of 33 rich countries with comparable data (OECD, 2021). Where school premises were closed, distance education (i.e. remote schooling) tended to replace in-school teaching, but schools and teachers both within and between countries were not equally equipped with the necessary tools and capabilities to ensure effective remote learning for all (OECD, 2020, 2021).

The Republic of Ireland experienced one of the harshest lockdowns in the world in response to the pandemic in 2020 (Hale et al., 2021). School premises were closed from 13 March 2020 until 30 June 2020, which is the end of the academic year for primary schools. At 141 school days, this was one of the longest school closures across rich countries at the time (Richardson et al., 2020). However, teaching and learning continued, albeit remotely, via digital communication and learning technologies (Symonds et al., 2020). Such an unexpected and rapid transition to remote schooling required considerable involvement from parents, especially those of younger children. This raised concerns about widening educational inequalities not only because parents differ in their capacity to support their children’s learning (Doyle, 2020), but also due to inequalities in children’s access to digital technologies at home (Armitage & Nellums, 2020). Children’s capacity to engage with remote schooling may also vary, and those with mental health and/or learning difficulties face particular challenges (Becker et al., 2020).

Children’s well-being at school matters to their emotional health (Kidger et al., 2012) and academic outcomes (Rumberger & Rotermund, 2012). Children from higher socio-economic status families tend to report higher levels of well-being at school (Loft & Waldfogel, 2021) and do better in tests of cognitive ability and/or academic achievement (Bradbury et al., 2019). Therefore, differences in children’s individual experiences of remote schooling during the COVID-19 lockdown may widen gaps in both well-being and achievement. This may not only affect children’s outcomes at present but have long-lasting consequences for their futures.

This study contributes to the wider debate on the social impacts of COVID-19 and the growing body of evidence on inequalities in children’s schooling during the pandemic by analyzing differences in primary school children’s engagement with remote schooling during the Spring 2020 lockdown in Ireland. It investigates whether children’s engagement with their remote schooling varied by personal and family characteristics, using data from the Children’s School Lives (CSL) surveys. The CSL is a nationally representative study of primary schools in Ireland, which collected information from children aged 8–9 years in May – August 2019 (“pre-Covid”, Wave 1) and in May – July 2020 (“Spring 2020 lockdown”, Wave 2). Our estimates are based on the analytic sample of nearly 400 children (from across 71 schools) who took part in both waves and have complete data on all the key variables.

### Engagement with Remote Schooling

Engagement is an important component of wellbeing (Seligman, 2018). It has been conceptualized as a multi-tiered, multi-faceted state of involvement in an activity (Skinner, 2016). Activities can be momentary and task specific, such as a mathematics problem issued by a classroom teacher to a student, or longer term and broader in scope, such as engaging in attending school over a school year (Symonds et al., 2021). Engagement has cognitive, emotional, and behavioural components (Fredricks et al., 2019). At the “macrolevel” (Sinatra et al., 2015) end of engaging over time with schooling as a complex activity, engagement’s emotional component is often represented as children’s emotional attitudes towards school, for example how much children like school, look forward to going to school, and enjoy being at school (Symonds & Hargreaves, 2016).

Emotional engagement with schooling is a particularly useful indicator of children’s overall experiences of the transition to remote schooling during the COVID-19 pandemic. Children’s emotional attitudes towards schooling develop over time as an outcome of their relationships with teachers and peers, emotional experiences in lessons, and the fit between their skills and interests and the curriculum (Symonds & Hargreaves, 2016). Therefore, children’s emotional engagement with remote schooling can serve as a top-level indicator of a wide range of meaningful interactions between children and their remote learning experience.

Emotional engagement with remote learning during the COVID-19 pandemic is being studied internationally by an increasing number of research teams. In Palestine, middle school students described their attitudes towards remote schooling as being formed out of their daily experiences of technology and connectedness with teachers, within a broader sociocultural system of familial norms and traditions (e.g. parental concerns about Internet safety and digital privacy) and structural inequalities related to access to learning resources, including digital inequalities (Khlaif et al., 2021). In the Philippines, a quantitative study of high school students uncovered that, on average, students felt that remote learning was discomforting and did not feel confident in learning online, and that their attitudes were less positive in classrooms where teachers felt less competent in teaching remotely (Salayo et al., 2020). Yet in Denmark, Skovgaard Jensen and Reimer (2021) found higher rates of liking school during the lockdown (when children were taught remotely), especially among lower socio-economic status students (i.e. those whose mothers did not have a tertiary degree). They used data from the Danish Student Wellbeing Survey, fielded both just before and during the spring lockdown. Other studies internationally have analysed teachers’ and parents’ perceptions on students’ emotional engagement. For example, in China parents described the inability of remote teaching to recreate the supportive relational atmosphere of the classroom and expressed concerns about academic progress for students with poor self-regulated learning once those students were outside of the physical classroom (Zhang, 2021).

On balance, this small but growing field of research has uncovered that the transition to remote learning during the COVID-19 pandemic has been detrimental for many children’s emotional engagement with school. However, this evidence was not generated about remote learning under typical circumstances. During the pandemic, children’s experiences of remote learning took place after a sudden transfer from in-person learning, and with teachers who might have had very little experience of remote teaching beforehand. Therefore, these studies represent children’s emotional engagement with a specific and untested form of remote learning during a highly unusual and challenging period.

### School Closures and children’s Learning

A growing literature shows adverse impacts of the COVID-19 pandemic on children’s learning. Using data from national examinations conducted before and after the lockdown, Engzell et al. (2021) found that primary school pupils in the Netherlands made little progress while learning remotely, with greatest learning losses among more disadvantaged students. Maldonado and De Witte (2020) documented significant learning losses for the 2020 class in all subjects tested in standardized assessments across Flemish schools in Belgium, with the largest losses in schools with lower socio-economic status student composition. At the start of the Autumn 2020/21 school term, primary school students across the United Kingdom (UK) lost an estimated two months of progress in reading and more than three months in mathematics, on average, compared to the results in previous years (Renaissance Learning & Education Policy Institute, 2021b). Cattan et al. (2021) found that total learning hours among school children in England were about two hours lower in April–May 2020 (during the full closures) and June–July 2020 (during partial re-opening) than before the pandemic.

Given the well-documented socio-economic inequalities in children’s learning (Cooper & Stewart, 2020; Cunha & Heckman, 2007), the literature on the potential impacts of the pandemic expected school closures to affect the poorest children most (Armitage & Nellums, 2020). This would be in line with the “summer learning gap” research (see Alexander et al., 2016), which shows that children’s educational achievement tends to decline over the summer break, especially for those from disadvantaged backgrounds. Yet remote schooling is different from summer holiday school closures because children are expected to learn at home, primarily using digital platforms and technologies. Children from less advantaged households are predicted to do so less effectively because they tend to have poorer access to digital technologies, internet connectivity and suitable spaces to study at home, while being at a higher risk of income poverty and food insecurity (Van Lancker & Parolin, 2020). Less educated parents may also struggle to provide the same level of support for children’s home learning as their more highly educated peers. Thus, school closures in Spring 2020 in the United States (US) were estimated to result in substantial learning losses, particularly for those already behind (Kuhfeld et al., 2020a). Based on aggregate data from school assessments at the beginning of the 2020/21 school year in the US, Kuhfeld et al., (2020b) showed that this was indeed the case for mathematics but not for reading where the results were better than expected. However, the study could not detect differences by socio-economic status because more disadvantaged students were less likely to take part in the assessments.

### Socio-Economic Differences in Remote Learning

There is mounting evidence on the socio-economic inequalities in children’s remote schooling. Much of it is coming from UK-based studies employing different methodologies and data. Combining pre-pandemic data from the UK Time Use Survey and new data collected during the lockdown online (April–May 2020), Andrew et al. (2020) found that primary school children in higher-income households in England spent significantly more time on learning activities than their peers in lower-income households during the Spring 2020 lockdown, even though there were no differences in learning time before the pandemic when children learned at school. The study also documented substantial socio-economic inequalities in access to home-learning resources, both those provided by schools (e.g. online classes) and in the home (e.g. computer; own dedicated study space). A related study by Cattan et al. (2021) using data from two waves (April – May and June – July) of the same survey found that children from more socio-economically advantaged families were more likely to return to face-to-face schooling during the partial re-opening phase and to increase their learning hours more when they did so. Meanwhile, analyses of data from the first wave of the COVID-19 supplement to the UK Longitudinal Household Survey (“Understanding Society”) showed that school-aged children from lower socio-economic backgrounds spent less time on home learning activities than their more privileged peers during the April 2020 lockdown (Bayrakdar & Guveli, 2020; Eivers et al., 2020; Pensiero et al., 2020). The extent of children’s learning loss in 2019/20 and any catch-up learning in 2020/21 across the UK differed by family social class and region (Renaissance Learning & Education Policy Institute, 2021a).

Similar patterns are documented in other rich countries. Evidence from the Netherlands (Bol, 2020) suggests that more privileged families were able to dedicate more time and resources to home schooling during the first lockdown. A study of 1415 German upper-secondary students in the academic track (Gymnasium) by Dietrich et al. (2020) found substantial socio-economic inequalities in the number of hours devoted to remote learning during the Spring 2020 school closures. Using information on internet search data across the US during the period when schools closed for the first time during the COVID-19 pandemic, Bacher-Hicks et al. (2021) found larger increases in search intensity for online learning resources in higher-income areas.

A small body of research indicates similar patterns for Ireland. A survey of secondary level school leaders carried out in Ireland during the Spring 2020 lockdown showed that student engagement with remote learning was greater in catchment areas with higher educational attainment (Mohan et al., 2021). A survey of principals and teachers in the Children’s School Lives study found contrasting rates of participation with remote learning among primary school children, with 20% of teachers of third-class children and 10% of principals indicating that just half the children in their class/school was accessing it (Symonds et al., 2020, p. 56). In a nationally representative “Social impact of COVID-19” survey conducted in August 2020, two-fifths (41%) of adults with children in primary school and nearly half (46%) of those with children in secondary school said that Spring 2020 school closures had a major or moderate negative impact on their children’s learning (Central Statistics Office, 2020). An online survey of parents of 12-year-old children who are members of the 2008 birth cohort of the “Growing Up in Ireland” study, carried out in December 2020, showed that only half of the children have always had a quiet place to study, three-quarters (74%) have always had access to a suitable computer, and one-fifth (19%) have always had access to online classes during school closures (Murray et al., 2021). However, to date, no evidence is available on how Irish children themselves felt about remote schooling during the pandemic and whether this differed by their personal characteristics or family background.

This literature suggests several hypotheses. We expect to observe higher levels of engagement with remote schooling in primary school children who 1) come from higher socio-economic status families; 2) have better resources for remote learning; and 3) exhibited higher levels of engagement with schooling before the pandemic.

## Methods

### Participants and Procedures

We used data from the first two waves of the ongoing mixed methods Children’s School Lives (CSL) study (https://cslstudy.ie). CSL is a nationally representative longitudinal study of primary schools in Ireland, commissioned by the National Council for Curriculum and Assessment and conducted by the School of Education at University College Dublin. CSL involves two cohorts of children: Cohort A (4/5-years-old in 2019) and Cohort B (8/9-years-old in 2019). Each cohort was drawn from a random sample of primary schools in Ireland. The study is ongoing and follows up the same children once per year until 2025. In this paper we analysed the existing data from the first two waves of the study. Fieldwork for Wave 1 took place over the period May – July 2019 via structured questionnaires delivered in classrooms by trained fieldworkers, while Wave 2 fieldwork was carried out using online questionnaires in May – July 2020. See Devine et al. (2020) and Symonds et al. (2020) for further details of the CSL objectives and methodology.

This paper focuses on Cohort B children, and their experiences of remote schooling during the first COVID-19 lockdown in Ireland. Although the wider CSL study includes children, parents, teachers, and principals, we used child-reported data from Waves 1 and 2 as well as teacher-reported information from Wave 1. The parents of Cohort B children were not interviewed in the Wave 1 survey, and few took part in Wave 2. We did not use parent data given that our outcome variable was measured at Wave 2 and only a small number of parents (N = 170) gave data that could be matched to children with valid Wave 1 and Wave 2 data. This meant that there were many missing observations on all the key variables for the parent sample.

The Wave 1 Cohort B sample included 2062 children (from across 97 schools) for whom parents gave consent to take part in the survey, but only 1879 of these children gave their assent (91%) and responded to the child questionnaire. Of these, 725 children (from across 89 schools) took part in the Wave 2 data collection, but only 540 filled out the questionnaire. The analytic sample in this study consists of 374 children who had complete data on all the key variables from both waves.

We carried out a series of sensitivity analyses. First, we determined the extent to which the Wave 1 sample is similar to the cohort of children who were 9 years old in 2017–2018 based on comparable variables from the nationally representative “Growing Up in Ireland” study. Second, to establish the extent to which attrition between the two waves was non-random, we compared the two-wave analytic sample to the Wave 1 sample on key Wave 1 characteristics (see Table 1). As a robustness check, we then estimated a structural equation model using the maximum likelihood with missing values estimator (which utilises all the available information rather than listwise deleting any observation with a missing value on at least one variable) and compared the findings to the main models that were based on listwise deletion.

**Table 1:** Descriptive statistics

	Mean	SD	Min	Max	N	Mean	SD	N
	Panel 1					Panel 2		
**Wave 1**	Whole sample					Analytic sample		
Girl	0.51	0.50	0	1	2053	0.52	0.50	380
Lives with both parents	0.85	0.36	0	1	1903	0.93***	0.26	380
Family affluence scale	5.71	1.32	0	7	1795	5.89*	1.22	380
Other language than English at home	0.15	0.36	0	1	1803	0.11**	0.31	380
SDQ Peer Relationship Problems	0.98	1.53	0	8	1778	0.88	1.45	380
SDQ Conduct Problems	0.83	1.46	0	10	1778	0.70*	1.31	380
SDQ Emotional Symptoms	1.69	2.13	0	10	1778	1.48*	1.93	380
SDQ Hyperactivity-Inattention	2.99	2.92	0	10	1749	2.39***	2.44	380
I look forward to going to school	3.23	1.26	1	5	1860	3.29	1.19	379
I like being in school	3.36	1.28	1	5	1853	3.38	1.18	377
I wish didn’t have to go to school (reverse coded)	3.31	1.45	1	5	1849	3.40	1.38	376
I like many things about school	3.62	1.22	1	5	1842	3.73*	1.10	375
*School engagement scale*	100.00	10.00	77.63	115.14	1825	100.59	9.50	374
**Wave 2**								
I look forward to home schooling	2.70	1.19	1	5	521	2.66	1.20	380
I wish I didn’t have to do home schooling (reverse coded)	2.60	1.25	1	5	521	2.58	1.22	380
I like doing home schooling	2.85	1.18	1	5	521	2.84	1.18	380
I like many things about home schooling	3.01	1.16	1	5	519	2.98	1.15	380
Home schooling is interesting and fun	3.07	1.12	1	5	521	3.03	1.12	380
*Home school engagement scale*	100.00	10.00	81.77	121.02	519	99.72	9.95	380
Does schoolwork on a computer	0.32	0.47	0	1	496	0.30	0.46	380
Can always get help with schoolwork if worried	0.59	0.49	0	1	537	0.60	0.49	380
School work is checked by teacher	0.59	0.49	0	1	534	0.58	0.49	380

Source: Children’s School Lives study (Cohort B Waves 1 and 2)

Notes: *** p < 0.001, ** p < 0.01, * p < 0.05 - from separate ordinary least squares regressions of each variable on an indicator of whether the respondent is in the analytic sample

### Variables

#### Engagement with Remote Schooling

We treat engagement with schooling as a latent construct based on a series of items, each of which may be measured with error. Engagement with remote schooling is measured using five child-reported items on satisfaction with schooling from Wave 2, adapted from Huebner’s (1994) Multidimensional Students’ Life Satisfaction Scale (MSLSS). Rowe et al. (2010) validated the MSLSS items as a component of a personal (rather than group-based) measure of classroom climate among primary school students, capturing the extent to which students like their classroom.

The CSL Wave 2 survey contains the following five questions:

1. I look forward to home schooling;
2. I like doing home schooling;
3. I wish I didn’t have to do home schooling;
4. I like many things about home schooling;
5. Home schooling is interesting and fun.

Responses were on a 5-point scale ranging from 1 “Never” to 5 “Always”. We reverse-coded Question 3 so that higher values would correspond to greater engagement with remote schooling. The five questions formed a highly reliable scale (alpha = 0.91) and loaded on one latent factor using principal component factor analysis, which explained 75% of the overall variation in the items. We standardized the resulting factor scores to a mean of 100 and a standard deviation of 10 to use as the outcome variable in the regression models.

#### Socio-Economic Status

We measured family SES using a set of child-reported variables from Wave 1: how many cars (none, one, two or more) and computers (none, one, two, three or more) the family has, as well as how often they went on holiday over the past year (not at all, once, twice or more). We summed responses to these questions into a scale that ranges from 0 to 7. This is a short version of the Family Affluence Scale (Currie et al., 1997; Torsheim et al., 2016) used in the Health Behaviour in School-aged Children survey, an international school-based study of children aged 11–15.

#### Resources for Home Learning

We use three child-reported Wave 2 items to measure children’s resources for home learning. The first item is an indicator of digital resources: whether the child does their schoolwork on a computer/laptop at home, as opposed to on a tablet or one’s own or a parent’s smart phone. These are asked as separate “yes/no” questions and are not mutually exclusive: a child may use all three types of devices for homework. We measure children’s resources by whether they use a computer or not because computers have emerged as an indispensable tool for home schooling during the pandemic (Goudeau et al., 2021), given their range of functions for school work such as word processing tools, design tools, Internet browsers, and video calling. Indeed, exploratory analyses showed that using a computer/laptop for schoolwork was associated with statistically significantly higher engagement with schooling, while doing schoolwork on a tablet or smartphone was not significantly associated with engagement.

The other two home learning resource items refer to remote schooling support that children can avail of: “If I am worried about falling behind in home school, I can get help” (scored on a 5-point scale from 1 “Never” to 5 “Always) and “My work in home school is checked by teacher” (no/yes/“isn’t checked at all”). We recoded responses to these questions into binary variables to identify those who say they can always get help if they are worried and those whose work gets checked by a teacher, respectively. We dichotomized responses to the “get help if worried” variable because the majority (60%) of the children said they could always get help and only 12% responded “rarely” or “never”, suggesting that the main difference was between those who could always get help and everyone else.

#### Other Predictors and Controls

We also used data on child satisfaction with schooling collected in Wave 1 to measure children’s engagement with schooling before the pandemic. Out of the five items measured at Wave 2, only four were available at Wave 1. There was no equivalent to “Home schooling is interesting and fun”. The remaining four items formed a reliable scale (alpha = 0.83) and loaded on one factor that explained 67% of their variation. We standardized the factor scores to a mean of 100 and a standard deviation of 10.

To account for variation in children’s behavioural and socio-emotional functioning before the pandemic, we used the teacher reported Strength and Difficulties Questionnaire (SDQ) sub-scales (Goodman, 1997). Finally, we controlled for the child’s gender, whether the child lives with both biological/adoptive parents (rather than with one parent or with a parent and a step-parent); and whether English was the main language spoken at home.

### Estimation

Since schools are the primary sampling units in the CSL survey, and children in the same school are likely to be more similar on key observable and unobservable characteristics than children randomly drawn from the population, we need to account for the multilevel structure of the data. The CSL school sample was drawn to be representative of the national population of primary schools, so a multilevel ‘random intercepts’ model where school-level residuals are assumed to be independent and identically distributed could be appropriate (Snijders & Bosker, 2012). However, estimating an ‘empty’ random intercepts multilevel model of engagement with remote schooling in the analytic sample of 374 children from across 71 schools (with between 1 and 19 children per school) produced an intra-class correlation (ICC) of just 1%. This means that only 1% of the total variation in the outcome is due to between-school effects and nearly all the variation in schooling engagement is among children within schools. A random intercepts model with all the predictors included does not alter the unexplained between-school variance, while a likelihood ratio test that compares this model to a single-level linear regression (without a school-level residual) does not detect a statistically significant difference. This suggests that the school residual does not need to be modelled as a random parameter.

Instead, we estimated the following linear regression with school fixed effects that controls for unobserved differences between schools by including school dummies as parameters to be estimated (see Models 1–3 in Table 2):

**Table 2:** Linear regression of engagement with remote schooling (school fixed effects)

	Model 1	Model 2	Model 3
Wave 1: Girl	3.65**	3.28*	2.56*
Wave 1: Lives with both parents	3.44	2.48	2.63
Wave 1: Family affluence scale	0.38	0.19	0.19
Wave 1: Other language than English at home	0.70	1.09	0.02
Wave 1: SDQ Peer Relationship Problems	0.88	0.72	0.80
Wave 1: SDQ Conduct Problems	0.06	0.30	0.26
Wave 1: SDQ Emotional Symptoms	−0.11	−0.10	−0.17
Wave 1: SDQ Hyperactivity-Inattention	−0.55*	−0.59*	−0.50
Wave 2: Does school work on a computer		3.11*	2.94*
Wave 2: Can always get help with schoolwork if worried		2.90*	2.95**
Wave 2: School work is checked by teacher		3.73**	3.16*
Wave 1: Engagement with schooling			0.24***
Intercept	94.26***	91.13***	69.01***
N (children)	374	374	374
N (schools)	71	71	71
R-squared	0.24	0.30	0.33
Adjusted R-squared	0.04	0.10	0.14

Source: Children’s School Lives study (Cohort B Waves 1 and 2)

Notes: *** p < 0.001, ** p < 0.01, * p < 0.05

Estimates of school ID dummies in models 1–3 are omitted for clarity

$${y}_{ij}={\beta}_0+\sum_{j=1}^{J-1}{\gamma}_j{\alpha}_j+{\beta}_1{x}_{1 ij}+\dots +{\beta}_k{x}_{kij}+{\epsilon}_{ij}$$

where *y*_*ij*_ is the remote schooling engagement score for child *i* in school *j*; there are *k* explanatory variables *x* that vary across children and schools; *α*_*j*_ are a series of (j-1) school dummies, and *ϵ*_*ij*_ is the error term for child *i* in school *j.* This means that the *β* coefficients can be interpreted as ceteris paribus differences in mean schooling engagement *in any given school*.

We entered predictors sequentially to test three sets of inter-related hypotheses. Children reported higher levels of engagement with online schooling if they 1) came from higher socio-economic status; 2) had better resources for remote learning; and 3) exhibited higher levels of engagement with schooling before the pandemic. First we adjusted for the family affluence score and other personal and family characteristics measured at Wave 1: the child’s gender, teacher reported SDQ sub-scale scores, family structure, and the language spoken at home (Model 1). We then added three measures of resources for learning (Model 2) and, finally, the school engagement score from Wave 1 (Model 3). Prior engagement was added last to check if resources for learning would still be significantly associated with remote schooling engagement even after controlling for prior schooling engagement.

We also included alternative model specifications as robustness checks. First, we re-estimated our final model (Model 3) without school fixed effects but with the standard errors adjusted for clustering at the school level (see Model 4 in Table 3). This helps ascertain which characteristics are robust to model specification, i.e. whether the findings are similar regardless of whether unobserved heterogeneity between schools is accounted for. Second, we estimated a structural equation model of latent engagement with schooling using a maximum likelihood with missing values estimator (see Model 5 in Table 4). Instead of listwise deleting all observations with a missing value on any of the variables in the model, this approach preserves as much information as possible from observations with missing values. This specification helps establish if our main results are influenced by non-random selection into the analytic sample. All models were estimated in Stata 16.

**Table 3:** Linear regression of engagement with remote schooling (cluster robust standard errors)

	Model 4
Wave 1: Girl	1.09
Wave 1: Lives with both parents	2.99*
Wave 1: Family affluence scale	−0.03
Wave 1: Other language than English at home	−1.08
Wave 1: SDQ Peer Relationship Problems	0.68
Wave 1: SDQ Conduct Problems	0.33
Wave 1: SDQ Emotional Symptoms	0.09
Wave 1: SDQ Hyperactivity-Inattention	−0.35
Wave 2: Does school work on a computer	3.72***
Wave 2: Can always get help with schoolwork if worried	3.02**
Wave 2: School work is checked by teacher	2.75**
Wave 1: Engagement with schooling	0.24***
Intercept	68.16***
N (children)	
N (schools)	
R-squared	0.18
Adjusted R-squared	0.15

Source: Children’s School Lives study (Cohort B Waves 1 and 2)

Notes: *** p < 0.001, ** p < 0.01, * p < 0.05

**Table 4:** Structural equation model of latent engagement with remote schooling (maximum likelihood with missing values estimator), standardized coefficients

	Model 5
Wave 1: Girl	0.03
Wave 1: Lives with both parents	0.07
Wave 1: Family affluence scale	0.03
Wave 1: Other language than English at home	−0.04
Wave 1: SDQ Peer Relationship Problems	0.10
Wave 1: SDQ Conduct Problems	0.06
Wave 1: SDQ Emotional Symptoms	0.03
Wave 1: SDQ Hyperactivity-Inattention	−0.09
Wave 2: Does school work on a computer	0.18***
Wave 2: Can always get help with schoolwork if worried	0.18***
Wave 2: School work is checked by teacher	0.12**
Wave 1: (Latent) engagement with schooling	0.27***
N (children)	2072
N (schools)	98
Root mean squared error of approximation (RMSEA)	0.01
Coefficient of determination (CD)	0.89

Source: Children’s School Lives study (Cohort B Waves 1 and 2)

Notes: *** p < 0.001, ** p < 0.01, * p < 0.05. Standard errors adjusted for clustering at the school level. Variances, covariances, and factor loadings are not reported

## Results

Table 1 reports descriptive statistics separately for the sample of children who responded to any of the questions in Wave 1 or Wave 2 (Panel 1), and for the analytic sample of nearly 400 children who had non-missing observations on all the Wave 1 and Wave 2 measures used in the analysis (Panel 2). The N column in each panel shows the number of valid observations. For example, there is gender information for 2053 children in Wave 1 and for 380 children in the analytic sample.

The Wave 1 sample is equally split between girls and boys. The vast majority (85%) lived with both parents in 2019. This is similar to the two-parent family rate of 85% among 9-year-old-children in the large nationally representative “Growing Up in Ireland” (GUI) 2008 birth cohort in 2017–18 (McNamara et al., 2021, p. 28). A non-trivial minority of CSL children (15%) reported speaking a language other than English at home. This is consistent with 20% of mothers and 17% of fathers of the GUI study children having been born outside Ireland or Britain (Williams et al., 2010, p. 36).

The means and the standard deviations (in brackets) of the teacher-reported SDQ sub-scales are also in line with GUI at a similar age (McNamara et al., 2021, p. 84): peer relationship problems (1.0 [1.5] in CSL vs 1.0 [1.6] in the GUI); conduct problems (0.8 [1.5] in CSL vs 0.7 [1.4] in GUI); emotional symptoms (1.7 [2.1] in the CSL vs 1.6 [2.1] in the GUI); and hyperactivity (3.0 [2.9] in CSL vs 2.7 [2.7] in GUI). This indicates that the Wave 1 sample is broadly representative of the national population of 9-year-olds.

The sample of children who took part in both waves and have non-missing data on all key variables differ from the Wave 1 sample on several characteristics. The following predictors, all measured at Wave 1, are associated with analytic sample membership: living with both parents (p < 0.001), higher scores on the family affluence scale (p < 0.05), speaking English at home (p < 0.01), and lower scores on the teacher reported SDQ emotional problems sub-scale (p < 0.05), conduct problems sub-scale (p < 0.05), and the hyperactivity-inattention sub-scale (p < 0.001). This suggests that children from less socio-economically privileged families and children with emotional and behavioural problems were less likely to remain in Wave 2 and to have valid information on all the key Wave 2 measures. However, Table 1 shows that there were no statistically significant differences in the analytic sample membership across the variables measured in Wave 2 only.

This suggests that, while there may be positive self-selection into Wave 2 participation, those who took part in Wave 2 but had missing data on some of the Wave 2 measures are not statistically significantly different from those who had complete data on all the Wave 2 measures. As a robustness check, we re-estimated our regressions as structural equations models using the maximum likelihood with missing values (mlmv) estimator in Stata 16 (see Model 5 in Table 4). The results were substantially the same as in the main models.

Table 2 shows the school fixed effects linear regression estimates of children’s engagement with remote schooling (Models 1 — 3). Model 1 includes the family affluence scale and controls for the child’s gender, the language spoken at home, whether the child lived with both parents and the teacher reported SDQ sub-scale scores from Wave 1. There is no statistically significant association between the family affluence scale and engagement with remote schooling, everything else being equal. However, girls scored 3.7 points higher on the engagement with remote schooling scale (p < 0.01), on average. Children with higher hyperactivity-inattention scores reported lower engagement with remote schooling (p < 0.05), on average.

Model 2 adds measures of resources for remote learning available to children, measured at Wave 2. Children who did their schoolwork on a computer scored 3.1 points higher on the engagement with remote schooling scale, on average (p < 0.01), than those who did not. Children who said they can always get help with schoolwork if they are worried about it scored 2.9 points higher, on average (p < 0.05), than those who said they can get help sometimes or less frequently. Everything else being equal, children who said their schoolwork was checked by a teacher scored 3.7 points higher (p < 0.01). These are substantively large differences, given the standard deviation of schooling engagement of 10 points.

Model 3 adds a Wave 1 measure of school engagement. Everything else being equal, a one-point difference in school engagement before the pandemic is associated with 0.24 points (p < 0.001) higher engagement with remote schooling during the lockdown. This is a substantively large effect: a one standard deviation difference in school engagement at Wave 1 is associated with 0.23 standard deviations higher remote schooling engagement at Wave 2. Notably, controlling for this measure of pre-pandemic school engagement does not alter the coefficients of key Wave 2 predictors: having a computer for schoolwork, having someone to help with schoolwork, and having schoolwork checked by a teacher. However, the hyperactivity-inattention coefficient is no longer statistically significantly different from zero, suggesting that its effect in Model 2 was confounded by schooling engagement at Wave 1.

In all three models, girls tended to report higher levels of engagement with remote schooling than boys, all else being equal. Controlling for learning resources and prior school engagement (Model 3), girls scored 2.7 points more, on average (p < 0.05). This is a quarter of a standard deviation difference. We tested for a potential interaction effect between the family affluence scale and gender, but the interaction term was not statistically significantly different from zero.

Models 2 and 3 account for about a third of the variation in engagement with remote schooling. However, after adjusting for the number of predictors in the model, which include 71 school ID dummies, this falls to about one-tenth of the variation accounted for. This suggests that there are differences in remote schooling engagement due to unobserved differences between schools.

### Robustness Checks

To check the extent to which our findings are robust to alternative ways of dealing with the fact that children cluster within schools, we re-estimated Model 3 without school fixed effects. Instead, we adjusted the standard errors for clustering at the school level (Model 4 in Table 3). The coefficients on having a computer for schoolwork, having someone to help with schoolwork, and having schoolwork checked by a teacher, as well as prior engagement with schooling, are similar to those in Model 3, albeit somewhat larger and/or more precisely estimated. There are differences in the coefficients of control variables, however. There is no longer a statistically significant gender difference in remote schooling engagement, while living with both parents is now statistically significantly (p < 0.05) associated with higher remote schooling engagement.

Because a non-trivial proportion of children did not take part in Wave 2, we re-estimated Model 3 as a structural equations model using the maximum likelihood with missing values estimator (Model 5). While this approach preserves as much information as possible from observations with missing values, any missing values are assumed to be missing at random: they are either randomly dispersed throughout the data or the probability of being missing depends on other variables in the model. Since attrition between waves 1 and 2 was significantly associated with family affluence, family structure, language spoken at home, and teacher reported SDQ scores measured at Wave 1, it is important to control for these variables. Engagement with (remote) schooling is treated as a latent construct that is measured with four (five) observed indicators. The model uses information from all 2072 children across 98 schools The goodness-of-fit statistics indicate that the model fits the data very well (RMSEA<0.05 and CD = 0.89).

The findings are qualitatively similar to those from the regression models that are based on listwise deletion of all observations with at least one missing value (Model 3 in Table 2). Doing schoolwork on a computer, being able to get help with schoolwork if worried, having schoolwork checked by a teacher, and having higher schooling engagement before the pandemic are all statistically significantly associated with higher remote schooling engagement, ceteris paribus. The family affluence score is not significantly associated with remote schooling engagement and nor are any of the controls.

## Discussion and Conclusion

School closures have been one of the key mitigation measures in response to the COVID-19 pandemic in 2020, but their effects on children were predicted to be negative and inequitable (Armitage & Nellums, 2020; Van Lancker & Parolin, 2020). This study analyzed primary school children’s emotional engagement with remote schooling during the Spring 2020 lockdown in Ireland. It is a useful indicator of children’s overall experiences of learning during the unprecedented period of a rapid transition from face-to-face to remote schooling across Ireland. Although we do not have data on children’s academic achievement during the lockdown, engagement with remote schooling is likely to matter for the quality of their learning because engagement with schooling matters to educational attainment (Rumberger & Rotermund, 2012).

We estimated a series of school fixed effects regressions using unique data from the Children’s School Lives (CSL) surveys. The CSL is a unique, nationally representative study of primary schools in Ireland, which collected information from children aged 8–9 years in May – August 2019 (Wave 1) and in May – July 2020 (Wave 2). Our estimates are based on the analytic sample of nearly 400 children (from across 71 schools) who took part in both waves and had complete data on all the key variables. Our dependent variable was measured in Wave 2 using five child-reported items on emotional engagement with remote schooling. It captures the extent to which students liked their remote schooling.

At the outset of the study, we posited three hypotheses informed both by the vast literature on socio-economic status differences in children’s learning and experiences of schooling as well as on the emerging body of evidence on the consequences of the COVID-19 pandemic for children’s education. We expected to observe higher levels of engagement with remote schooling in primary school children who 1) come from higher socio-economic status families; 2) have better resources for remote learning; and 3) exhibited higher levels of engagement with schooling before the pandemic.

We measured socio-economic status using a short version of the Family Affluence Scale (Currie et al., 1997; Torsheim et al., 2016) based on children’s reports about cars, computers and family holidays at Wave 1. Children’s resources for home learning were measured using three child reported variables from Wave 2: whether the child does their schoolwork on a computer/laptop at home; whether the child can always get help if worried about falling behind in home school; and whether the child’s home schoolwork is checked by a teacher. We used four child-reported items on emotional engagement with schooling from Wave 1 as a measure of pre-pandemic engagement with schooling.

Surprisingly, and contrary to our first hypothesis, we did not find significant socio-economic differences in children’s engagement with remote schooling. There are several potential explanations for this finding. It could be that children were all equally impacted by the pandemic, regardless of socio-economic background. For example, Skovgaard Jensen and Reimer (2021) found higher rates of liking school during the lockdown in Denmark, particularly among lower socio-economic status students. It could also be that the well-documented SES differences in children’s in-person learning do not translate as readily into remote schooling because children whose parents work full-time do not gain as much support with home schooling as their peers whose parents lost their jobs or were on furlough during the pandemic. Villadsen et al. (2020) showed that parents who were working during the first lockdown in the UK spent less time on homeschooling than those who were not working. Hupkau et al. (2020) found that in the UK, children whose fathers’ earnings reduced to zero because of the pandemic were less likely to get additional paid resources but more likely to receive additional parental help of about 30 min per day. Del Bono et al. (2021) found no SES differences in parental time spent on home schooling in the UK.

It could also be that the short version of the family affluence scale as reported by children aged 8–9 is not a valid measure of family SES in Ireland. Information about the number of computers, cars and family holidays may not be sufficient to capture differences in family economic and social resources. The family affluence scale was not statistically significantly associated with the indicators of resources for home learning.

Yet our analysis is also limited by lower socio-economic and ethnic background variation in the analytic sample compared with the Wave 1 sample (see Table 1). Therefore, we do not necessarily have sufficient evidence to discount the hypothesis that there may be important SES differences in children’s emotional engagement with remote schooling. Qualitative data analysis of a selected case study sample of schools in CSL highlights SES differences in remote school experiences from the perspectives of teachers, principals and parents, as well as national survey data of teacher and principal reports (Symonds et al., 2020).

However, our results supported our second hypothesis. Children with greater resources for home schooling reported higher levels of engagement, everything else being equal. Having a computer or a laptop for schoolwork is significantly positively associated with greater engagement with schooling. So is having someone to help with schoolwork if the child is worried about falling behind and having schoolwork checked by a teacher. These estimates are robust to alternative specifications (i.e., school fixed effects; school-cluster adjusted standard errors; structural equation model using the maximum likelihood with missing values). This corroborates the results from the CSL surveys of schools, where more than two-thirds (68%) of teachers and three-fifths (61%) of principals reported that “any lack of participation in their classroom/school was because parents lacked access to digital technologies at home” (Symonds et al., 2020, p. 57). Our findings are consistent with the emerging literature that points to the paramount importance of adequate digital technologies at home during the pandemic (Lucas et al., 2020) as well as access to help with remote schooling, whether from parents or schools (Agostinelli et al., 2020; Cattan et al., 2021).

Finally, we expected that children who were more engaged with school before the pandemic would report higher levels of emotional engagement with remote learning. We have sufficient evidence to support this hypothesis. After controlling for home learning resources, socio-demographic characteristics and socio-emotional and behavioural difficulties measured at Wave 1, children who reported higher engagement with schooling before the pandemic were more engaged with remote schooling during the lockdown. Engagement with (remote) schooling was treated as a latent construct measured with multiple items. Importantly, controlling for prior engagement did not alter the findings with respect to home learning resources.

In line with the literature on gender differences in school engagement and academic performance (Johnson et al., 2001; Martin, 2007; Wang et al., 2011), we found significantly higher remote learning engagement levels in girls than in boys. However, the gender difference is not robust to model specification. It is only statistically significant in school fixed effects (i.e., within-schools) models. This suggests that *in any given school*, girls tend to report higher levels of engagement, even after controlling for a range of other relevant predictors, but the schools with a higher share of girls do not necessarily have higher levels of engagement, on average.

We also found that children’s ability to engage with remote schooling varied with their socio-emotional and behavioural functioning. Children who had higher scores on the hyperactivity sub-scale of the Strengths and Difficulties Questionnaire (SDQ) reported lower engagement levels with remote learning even after controlling for children’s home resources for remote schooling. This is in line with the studies that underscore differences in children’s individual capacity to engage with remote schooling effectively (Becker et al., 2020) and highlights the difficulties that children with special educational needs have faced during lockdowns (Waite et al., 2021). However, the association disappeared when we controlled for prior engagement with schooling. Since prior engagement is negatively correlated with each of the SDQ sub-scales, this indicates that children with hyperactivity-inattention difficulties struggled to engage with schooling both before and during the pandemic.

Although participation in Wave 2 of the survey was higher for children living with both parents and those who speak English at home, neither family structure nor language spoken at home were significantly associated with remote schooling engagement in the main model. Children living with both parents reported higher levels of school engagement, on average, but only in the model with the standard errors adjusted for school-level clustering and not in the school fixed effects models. This indicates that in any given school, children from two-parent and single-parent or step families do not differ in their average levels of engagement. Meanwhile, speaking a language other than English at home is not statistically significantly associated with schooling engagement in any of the models, everything else being equal.

Our study has important limitations. First, our analytic sample was relatively small because we had to limit the analyses to children who had valid responses on all key variables during both the first and second wave of data collection. Moreover, children were significantly more likely to take part in Wave 2 if they came from higher SES families, lived with both parents, spoke English at home and rarely or never came to school without homework, all measured at Wave 1. However, re-estimating our regressions in a structural equation modelling framework using all the available information (without listwise deletion) did not alter the estimates from the main model. Second, we do not have repeated measurements on the same variables in both waves, so we are unable to account for unobserved heterogeneity among children (using individual-level fixed effects regressions). Furthermore, our findings cannot necessarily be generalized to all instances of remote schooling. Our results are based on data collected during the first COVID-19 lockdown in Ireland in Spring 2020, at the time of a sudden transition to remote schooling that neither teachers, parents nor students had been prepared for. However, according to data from the UK collected during a longer span of the lockdown, children and parents do not necessarily ‘settle in’ to home learning as the lockdown progresses (Cattan et al., 2021).

Nonetheless, this is the first study to analyze the impact of the COVID-19 pandemic on children’s experiences of remote schooling in Ireland, which went through one of the strictest Spring lockdowns among all European countries. Using unique data from Ireland, our study highlights the importance of access to resources, both in terms of technology and parental/teacher support, to improve children’s engagement with online learning during periods of school closures. A higher emotional engagement with online learning is likely to lead to greater involvement with remote schooling, which in turn, would be expected to lead to better educational outcomes for children.
